# Temporal Regulation of GABA_A_ Receptor Subunit Expression: Role in Synaptic and Extrasynaptic Communication in the Suprachiasmatic Nucleus

**DOI:** 10.1523/ENEURO.0352-16.2017

**Published:** 2017-05-01

**Authors:** James C. Walton, John K. McNeill, Khallyl A. Oliver, H. Elliott Albers

**Affiliations:** Neuroscience Institute and Center for Behavioral Neuroscience, Georgia State University, Atlanta, GA 30303

**Keywords:** GABRD, GABRG2, entrainment, tonic inhibition, phasic inhibition, circadian, GABA

## Abstract

Recent molecular studies suggest that the expression levels of δ and γ2 GABA_A_ receptor (GABA_A_R) subunits regulate the balance between synaptic and extrasynaptic GABA neurotransmission in multiple brain regions. We investigated the expression of GABA_A_δ and GABA_A_γ2 and the functional significance of a change in balance between these subunits in a robust local GABA network contained within the suprachiasmatic nucleus of the hypothalamus (SCN). Muscimol, which can activate both synaptic and extrasynaptic GABA_A_Rs, injected into the SCN during the day phase advanced the circadian pacemaker, whereas injection of the extrasynaptic GABA_A_ superagonist 4,5,6,7-tetrahydroisoxazolo(5,4-c)pyridin-3-ol (THIP) had no effect on circadian phase. In contrast, injection of either THIP or muscimol during the night was sufficient to block the phase shifting effects of light. Gene expression analysis of the whole SCN revealed different temporal patterns in GABA_A_δ and GABA_A_γ2 mRNA expression. When examined across all subregions of the SCN, quantitative immunohistochemical analysis found no significant variations in GABA_A_δ protein immunoreactivity (IR) but did find significant variations in GABA_A_γ2 protein-IR in hamsters housed in either LD cycles or in constant darkness. Remarkably, significant interactions in the ratio of GABA_A_δ:GABA_A_γ2 subunits between lighting condition and circadian phase occurred only within one highly discrete anatomical area of the SCN; a region that functions as the input for lighting information from the retina. Taken together, these data support the hypothesis that the balance between synaptic and extrasynaptic GABA_A_Rs determines the functional response to GABA, and that this balance is differentially regulated in a region-specific manner.

## Visual Abstract

**Figure:**
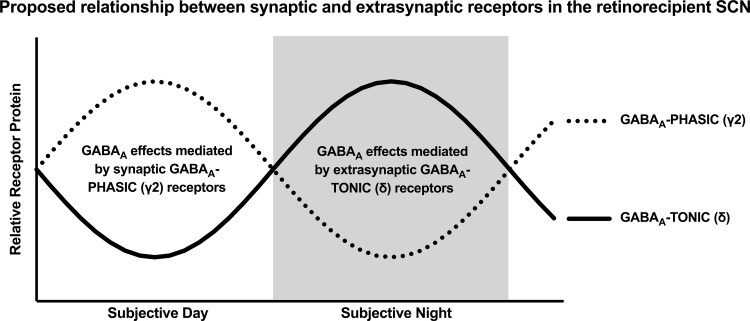

## Significance Statement

GABA neurotransmission is mediated primarily by GABA_A_ receptors (GABA_A_Rs). These receptors are composed of different combinations of five subunits that determine their pharmacological properties and subcellular location. Differences in the expression of GABA_A_Rs that contain the γ2 subunit versus those that contain the δ subunit may regulate the balance between synaptic and extrasynaptic GABA neurotransmission. We report here that expression of the γ2 and the δ subunits are differentially regulated within the circadian pacemaker in the suprachiasmatic nucleus (SCN) and provide evidence that the balance between synaptic and extrasynaptic GABA_A_Rs determine the functional response to GABA and that this balance is regulated in a site-specific manner within the SCN.

## Introduction

GABA, the primary inhibitory neurotransmitter in the brain, plays a key role in regulating the firing patterns of individual neurons and entire neural networks (Fritschy and Panzanelli, 2014). GABA_A_ receptors (GABA_A_Rs) are pentameric chloride channels comprised of three different proteins from 19 available subunits and are generally composed of two α, two β, and one γ, δ, or ε subunit (Olsen and Sieghart, 2009; Sigel and Steinmann, 2012; Fritschy and Panzanelli, 2014). Subunit composition determines their anatomic location and physiologic properties (Fritschy and Panzanelli, 2014).

α4, α5, α6, and δ subunits are found at peri- and extrasynaptic locations, whereas α1 and γ2 are found within the synapse (Farrant and Nusser, 2005). γ2 and δ subunits are mutually exclusive in receptor complexes (Araujo et al., 1998) and have different properties. δ GABA_A_Rs display tonic chloride conductance, do not readily desensitize, and are referred to as GABA_A_-TONIC receptors (Stell and Mody, 2002; Albers et al., 2017). γ2 GABA_A_Rs form perisynaptic clusters that then move into the synapse (Essrich et al., 1998; Danglot et al., 2003), where they modulate fast (phasic) conductance, rapidly desensitize following activation, have 50-fold lower GABA affinity, and are referred to as GABA_A_-PHASIC receptors (Stell and Mody, 2002; Albers et al., 2017). Although much is known about the diversity of GABA_A_Rs, little is known about their transcriptional regulation (Fritschy and Panzanelli, 2014) and even less about their specific roles in coregulating GABA networks.

The SCN in the anterior hypothalamus is the central circadian pacemaker that entrains an organism’s physiology and behavior to environmental light-dark (LD) cycles (Stephan and Zucker, 1972). The SCN provides the opportunity to study the network properties of GABA, because it contains a robust local GABA network with distinct inputs (e.g., light) and easily measured outputs (e.g., phase shift in circadian rhythms). Given that all or nearly all neurons within the SCN produce GABA as a neurotransmitter, it is likely that GABA has a fundamental role in circadian timekeeping (van den Pol, 1986; Moore and Speh, 1993; Castel and Morris, 2000; Albers et al., 2017). Indeed, GABA plays a major role in the ability of the circadian pacemaker to be reset by environmental stimuli. Muscimol, an agonist which activates GABA_A_Rs that contain either the γ2 or the δ subunit, injected into the SCN phase advances the circadian pacemaker during subjective day (Smith et al., 1989; Huhman et al., 1995; Mintz et al., 2002; Ehlen et al., 2006; Biello, 2009), mimicking the effects of nonphotic stimuli (e.g., locomotor activity; Mrosovsky et al., 1992; Mrosovsky, 1996). Diazepam, a benzodiazepine that acts at γ2 containing receptors similarly phase advances the clock during the subjective day (McElroy et al., 2009).

GABA_A_Rs are also critical in the phase resetting effects of light. Acute administration of muscimol into the SCN blocks the ability of light to induce phase delays in the early subjective night and phase advances during the late subjective night (Gillespie et al., 1996, 1997, 1999; Novak and Albers, 2004). Acute administration of the nonselective GABA_A_ antagonist bicuculline enhances light-induced phase delays during the early subjective night (Gillespie et al., 1996). More recently, the sustained activation of GABA_A_Rs has been found to be both necessary and sufficient to mediate the phase delaying effects of light during the early subjective night (Hummer et al., 2015). Taken together, it is clear that GABA_A_Rs play a fundamental role in determining how both light and nonphotic signals influence the phase of the pacemaker found within the SCN.

Despite the importance of GABA_A_Rs in regulating the phase of the circadian pacemaker, the role of GABA_A_Rs composed of different subunits is not well understood. Based on several studies, there is a consensus that α1, α2, β1, β2, and γ2 subunit mRNA or proteins are expressed in the SCN (Gao et al., 1995; O'Hara et al., 1995; Naum et al., 2001). To our knowledge, only one study has investigated GABA_A_δ in the SCN and reported it undetectable by Western blotting (O'Hara et al., 1995). Pharmacological evidence, however, indicates the presence of and a separate role in entrainment for both δ and γ2 GABA_A_Rs in the SCN (Ehlen and Paul, 2009; McElroy et al., 2009). The aim of this study was to investigate how the expression of GABA_A_δ and γ2 subunits varies within the SCN across circadian time (CT) to test the hypothesis that rhythms in GABA_A_-TONIC (δ) and GABA_A_-PHASIC (γ2) receptors and/or their ratio mediate the phase-dependent effects of GABA on the circadian pacemaker.

## Materials and Methods

### Animals and housing

Adult male Syrian hamsters (*Mesocricetus auratus*, 120–150 g) were purchased from Charles River Laboratories. On arrival, hamsters were singly housed in polycarbonate cages (23 × 43 × 20 cm) with corncob bedding, given ad libitum access to food (#5001; Lab Diet) and water, and maintained in 14:10 light:dark (LD) cycle for 7–10 d before any manipulation. The Department of Animal Resources at Georgia State University provided all animal husbandry. All procedures were approved by the Georgia State University Institutional Animal Care and Use Committee and were in compliance with guidelines established by the National Institutes of Health [Institute for Laboratory Animal Research (U.S.), 2011] and established by the Society for Neuroscience.

### Experiment 1: Effects of GABA_A_R subtype-specific agonists on phase resetting

Under isoflurane anesthesia, hamsters were stereotaxically implanted with a 26-ga guide cannula (PlasticsOne) aimed at the SCN region (AP +0.7 mm; ML +1.7 mm; 10° angle toward midline). Cannulae were anchored to the skull with bone screws and cranioplastic cement. Hamsters recovered a minimum of 7 d in LD, and were then given access to a running wheel (33 cm diameter; Techniplast) and placed in constant darkness (0:24 light:dark; DD). Running wheel activity rhythms were recorded remotely using VitalView software (Starr Life Sciences) and phase shifts in activity onsets were quantified using the linear regression method (Pittendrigh and Daan, 1976) and ClockLab software (Actimetrics). By convention, for nocturnal animals CT12 was defined as the time of activity onset. After a minimum of 10 d in DD, microinjections (200 nl, administered over a 20 s period) were given under dim red light with a 1.0-μl Hamilton syringe connected to a 33-ga needle that projected to a final depth of 7.8 mm below bregma. The needle remained in place for 20 s after the injection. The GABA_A_δ superagonist 4,5,6,7-tetrahydroisoxazolo(5,4-c)pyridin-3-ol (THIP) and the nonselective GABA_A_ agonist muscimol, purchased from Sigma, were dissolved in sterile 0.9% saline at concentrations of 110 and 11 mM, respectively (Ehlen and Paul, 2009; Hummer et al., 2015), immediately before injections. Although THIP is a superagonist at extrasynaptic (δ) receptors, it is only a partial agonist at synaptic (γ2) receptors at high concentrations (Hansen et al., 2001). Furthermore, THIP has very low affinity for native intrasynaptic γ2 receptors (Drasbek and Jensen, 2006), thus it is likely only affecting extrasynaptic GABA_A_δ receptors *in vivo*. For injections at CT6, hamsters were returned to their home cage in DD immediately after the injection. Injections at CT13.5 or CT19 were immediately followed by a 15-min 150 lux light pulse after which hamsters were returned to their home cages in DD. Hamsters with stable rhythms received an additional microinjection 10–14 d following the first treatment (to allow for stable reestablishment of the free-running rhythm) and were returned to running wheels in DD for another 10–14 d. No hamster received more than two injections. At the conclusion of testing, hamsters were killed by sodium pentobarbital overdose and then injected with ink to verify cannula placement. After histologic examination, hamsters with injection sites found to surround (within 500 μm), but not damage the SCN, were included in the analyses. It has been previously shown that drugs injected 500 μm or further from the SCN border do not phase shift the circadian pacemaker (Hummer et al., 2015) and that injections in a volume of 200 nl (the volume used in the present study) spread slightly less than a mm from the tip of the injection needle (Albers et al., 1990; Caldwell and Albers, 2003). The hamster SCN is ∼0.6 mm in the rostral-caudal plane, ∼0.3 mm in the mediolateral plane, and ∼0.6 mm in the dorso-ventral plane (Lydic et al., 1982). Because the hamster SCN lies ventral and not lateral to the third ventricle, and the SCN actually merge bilaterally midway along the dorsoventral axis, there is little barrier to the spread of drugs bilaterally. Indeed, it has been shown that injections using a volume of 200 nl diffuse bilaterally throughout the SCN (Gillespie et al., 1999; Paul et al., 2005). Taken together, these data suggest that injections within 500 μm of the SCN should diffuse throughout the bilateral SCN and for a short distance outside the borders of the nucleus.

### Experiment 2: GABA_A_R subunit gene expression in the SCN

After habituation to the animal facility, hamsters either remained in LD or were placed in DD and given access to running wheels as described in experiment 1 above. After 10 additional days in either LD or DD, hamsters were given a lethal overdose of sodium pentobarbital, decapitated, and brains were rapidly removed and placed in 2.5 ml of RNAlater (Ambion) then held at 4°C for one to two weeks before RNA extraction. Brains were collected at zeitgeber time (ZT)6, ZT13, and ZT19 from hamsters in LD, and at CT6, CT13, and CT19 from hamsters in DD. By convention for nocturnal animals ZT12 is the onset of activity, thus in the 14:10 LD cycle lights on occurred at ZT22 and lights off at ZT12. For ZT13, ZT19, and all DD time points, brains were collected under dim red light (<5 lux). After RNA stabilization in RNAlater, brains were then placed in a matrix and a 1.0 mm thick slice containing the SCN was collected onto a glass slide. SCN were then collected into 200 μl of Trizol (Ambion) using a 1.0-mm tissue punch. Individual SCN were homogenized in 1.0 ml Trizol using a sterile pestle and RNA was extracted following manufacturer’s protocol. RNA was washed twice with chloroform and precipitated with 100% isopropanol. The pellet was then washed twice with 75% ethanol, resuspended in 20 μl of water, and RNA concentration was determined using a NanoDrop 2000. Following extraction, 150 ng of total RNA was then reverse transcribed into cDNA using M-MLV (Promega) following the manufacturer’s protocol. Relative gene expression was quantified using an ABI 7500 FAST Real-Time system using Taqman Universal PCR master mix and the following universal two-step RT-PCR cycling conditions: 50°C for 2 min, 95°C for 10 min, followed by 40 cycles of 95°C for 15 s and 60°C for 1 min. The following primer/probe sets from Applied Biosystems were used: GABA_A_δ (ABI Mm01266203_g1), GABA_A_γ2 (ABI Rn00788325_m1), and 18s (4319413E). Relative gene expression for each sample run in duplicate was calculated by comparing to a relative standard curve and then standardized to 18S rRNA expression. Relative cDNA standards were generated using pooled hippocampal RNA extracts, which included tissue from animals at each CT point.

### Experiment 3: GABA_A_R subunit protein expression in the SCN

Hamsters were housed as described in experiment 2 above. At the same circadian and zeitgeber time points as described in experiment 2, hamsters were given a lethal overdose of sodium pentobarbital, followed by a transcardial perfusion with 100 ml of ice cold 0.1 M PBS, pH 7.4, then followed by 100 ml of freshly made ice cold 4% paraformaldehyde in 0.1 M PBS. Brains were removed and postfixed in 4% paraformaldehyde 0.1 M PBS at 4°C. After 12–16 h of postfixation, brains were placed in 0.1 M PB + 30% sucrose at 4°C. Once brains had sunk in the sucrose solution, they were then flash frozen in 2-methylbutane on dry ice, and held at −80°C until sectioning. Brains were sectioned at 40 μm on a cryostat, and three sets of serial coronal sections containing the SCN were collected into cryoprotectant and held at −20°C for immunohistochemical staining. A representative series of sections from each brain was then processed for either GABA_A_δ (Millipore catalog AB9752, RRID:AB_672966) or GABA_A_γ2 (Abcam catalog ab16213, RRID:AB_302324). Briefly, free floating tissue sections were rinsed three times in 0.1 M PBS + 0.1% Triton X-100 (PBST), blocked in 10% normal goat serum (NGS) in PBST for 30 min, and incubated in primary antibody (1:250 in PBST + 10% NGS) overnight at 4°C. Sections were then rinsed in PBST and incubated in secondary antibody (Jackson ImmunoResearch 111-065-003; 1:500 in PBS + 5% NGS) for 2 h at room temperature. After secondary incubation, tissue was rinsed in PBS, complexed with ABC (Avidin/Biotinylated enzyme Complex, Vector PK-6100), and developed with nickel 3,3’-diaminobenzidine (Ni-DAB; Vector SK-4100) according to the manufacturer’s protocols. Sections were then mounted onto chrome-gel subbed slides, dried, dehydrated in a graded ethanol series, cleared with xylenes, and coverslipped with Permount (Fisher). Immunohiostochemistry was yoked so that all tissue sections for each protein of interest were processed simultaneously allowing for direct comparisons of relative protein levels among groups.

Digital monochrome images were captured at 100× using a Zeiss Axioplan2 microscope fitted with a ProgRes SpeedXT core5 camera (JENOPTOK). All images used for protein quantification were taken in a single session without altering microscope or camera settings. For each representative series of brain sections, four images were captured representing the rostral, central anterior, central posterior, and caudal SCN as previously described in hamsters (LeSauter et al., 2002; Hamada et al., 2004). These regions correspond to those found in figures 23–25 of the golden hamster brain atlas (Morin and Wood, 2001). Using ImageJ, a region of interest (ROI) was defined that included the entire unilateral SCN. This ROI was then used to measure grayscale values of the SCN in each image. The grayscale value corresponds to the optical density of the DAB staining and thus is a measure of relative protein expression. Grayscale values were then inverted (255, measured value), so that higher numbers were indicative of relatively more protein-IR. Given that there is ongoing controversy about the functional neuroanatomical subdivisions of the SCN (reviewed in Moore et al., 2002; Lee et al., 2003; Morin and Allen, 2006; Morin, 2007; Evans, 2016; Evans and Gorman, 2016; Albers et al., 2017), and that GABA_A_ subunit distribution has been reported to vary across the rostro-caudal and dorso-ventral extent of the SCN (Gao et al., 1995; Belenky et al., 2003), we measured and analyzed protein expression in several different ways. First we analyzed the whole SCN by averaging the grayscale values of each ROI across the rostral-caudal extent, resulting in a single value for each whole SCN. Next, for a dorsal versus ventral anatomic division of the SCN, the initial ROI was further divided in half on the dorsal-ventral axis, and grayscale values were measured for each image and then averaged across the rostro-caudal extent of each SCN as described above, resulting in one dorsal and one ventral grayscale value. Finally, grayscale values were collected for each individual sub-ROI, resulting in eight grayscale values for each SCN (dorsal and ventral × four rostro-caudal divisions). All measurements were made by an observer blind to the experimental condition of the hamster.

Based on studies using genetic techniques in mice, GABA_A_ γ2 and δ appear to reciprocally regulate each other’s expression independent of receptor activity (Korpi et al., 2002; Wu et al., 2013). Thus, we also compared the relative protein-IR levels for the two GABA_A_R subtypes by comparing the relationship of their relative ratios (δ-IR:γ2-IR) across time points and lighting conditions. Although this ratio does not represent a direct measure of the absolute amounts of protein within the SCN, it does represent the relative change in the amounts of these proteins in relation to each other.

### Statistics

All statistical analyses were performed using SPSS 22.0 (IBM). Pharmacological data (experiment 1) were analyzed using one-way ANOVA (analysis of variance) with phase shift as the dependent variable and drug treatment as the independent variable. Significant ANOVAs were followed up with a Fisher’s LSD *post hoc* test. For experiment 2, gene expression data were also analyzed by one-way ANOVA with relative expression or expression ratio as the dependent variable and zeitgeber time or circadian time as independent variables. Significant ANOVAs were followed up with a Fisher’s LSD *post hoc* test. Gene expression data were also analyzed by independent samples *t* test with circadian phase as the independent variable. Protein-IR data were first analyzed using one-way ANOVA and independent samples *t* test as described above. To ascertain the anatomic location in the SCN of interactions between light regimen and circadian phase, protein-IR data were then analyzed by SCN anatomic subdivision using 2 × 2 MANOVA (multivariate analysis of variance) with grayscale value or expression ratio as the dependent variable and circadian phase and lighting condition as independent variables. To ascertain the effects of environmental lighting condition on GABA_A_ protein-IR, data were analyzed using an independent samples *t* test with lighting regimen (LD vs DD) as the independent variable. Finally, to ascertain the differences in GABA_A_ protein-IR between the dorsal and ventral SCN, a different independent samples *t* test was performed using these two factors as the independent variables. Differences were considered statistically significant at *p* ≤ 0.05. The numbers of animals used in each experiment are listed in Table 7.

## Results

### Experiment 1: Phase shifting effects of GABA_A_ agonists

**Figure 1.**
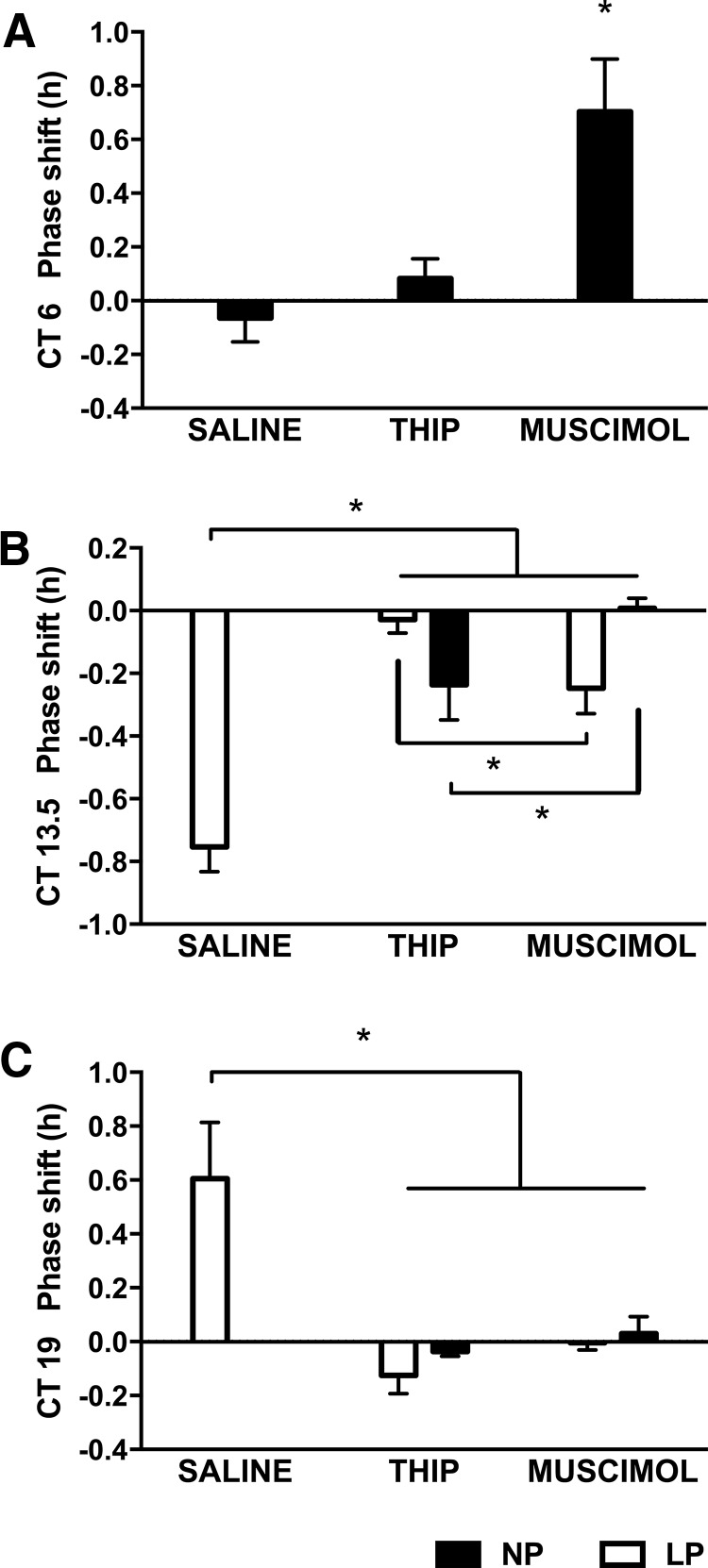
Extrasynaptic GABA_A_Rs contribute to the acute effects of GABA in the SCN during the subjective night but not during the subjective day. The nonselective GABA_A_-PHASIC/GABA_A_-TONIC agonist muscimol (2.2 nmol) phase advanced the pacemaker at CT6, whereas the GABA_A_-TONIC receptor superagonist THIP (22 nmol) had no effect (***A***). Both agonists were effective in blocking the phase shifting effects of a 15-min 150 lux light pulse during the subjective night (CT13.5 and CT19; ***B***, ***C***, respectively). THIP was more effective than muscimol at blocking photic phase delays at CT13.5 (***B***). In the absence of a light pulse, animals treated with THIP showed a small phase delay compared with those treated with muscimol at CT13.5 (***B***). Neither muscimol nor THIP had an effect on phase in the absence of a light pulse during the late subjective night (***C***). NP, no light pulse; LP, light pulse (150 lux, 15 min), *p ≤ 0.05. Statistics for all analyses in Table 1.

During the subjective day (CT6), the GABA_A_γ2/GABA_A_δ agonist muscimol induced a phase advance in circadian wheel running activity, whereas neither saline or the GABA_A_δ superagonist THIP had any effect on circadian phase (*F*_(2,21)_ = 8.544, *p* ≤ 0.05; Fig. 1*A*). During the subjective night, both THIP and muscimol blocked the phase delaying (CT13.5, *F*_(4,16)_ = 16.438, *p* ≤ 0.05; Fig. 1*B*) and phase advancing (CT19, *F*_(4,17)_ = 5.455, *p* ≤ 0.05; Fig. 1*C*) effects of a light pulse when compared with saline (Fig. 1). THIP was more effective than muscimol in blocking a light-induced phase delay during the early subjective night (CT13.5, *p* ≤ 0.05; Fig. 1*B*). However, in the absence of a light pulse at CT13.5, animals treated with THIP showed a small phase delay compared with those treated with muscimol (Fig. 1*B*; Table 1). Neither muscimol nor THIP had an effect on phase in the absence of a light pulse during the late subjective night (*p* > 0.05; Fig. 1*C*).

**Table 1.** Analysis of GABA_A_ active drugs on phase resetting

One-way ANOVA			
CT	*F* statistic	*p* value	
CT6	(2,21) = 8.544	*0.002	
CT13.5	(4,16) = 16.438	*0.000	
CT19	(4,17) = 5.455	*0.005	
LSD *post hoc*			
CT	Treatment vs	Treatment	*p* value
CT6	SALINE-NP	MUSCIMOL-NP	*0.001
		THIP-NP	0.488
	MUSCIMOL-NP	SALINE-NP	*0.001
		THIP-NP	*0.004
	THIP-NP	SALINE-NP	0.488
		MUSCIMOL-NP	*0.004
CT13.5	SALINE-LP	MUSCIMOL-LP	*0.000
		THIP-LP	*0.000
		MUSCIMOL-NP	*0.000
		THIP-NP	*0.000
	MUSCIMOL-LP	SALINE-LP	*0.000
		THIP-LP	*0.019
		MUSCIMOL-NP	*0.015
		THIP-NP	0.913
	THIP-LP	SALINE-LP	*0.000
		MUSCIMOL-LP	*0.019
		MUSCIMOL-NP	0.696
		THIP-NP	0.051
	MUSCIMOL-NP	SALINE-LP	*0.000
		MUSCIMOL-LP	*0.015
		THIP-LP	0.696
		THIP-NP	*0.036
	THIP-NP	SALINE-LP	*0.000
		MUSCIMOL-LP	0.913
		THIP-LP	0.051
		MUSCIMOL-NP	*0.036
CT19	SALINE-LP	MUSCIMOL-LP	*0.003
		THIP-LP	*0.001
		MUSCIMOL-NP	*0.007
		THIP-NP	*0.006
	MUSCIMOL-LP	SALINE-LP	*0.003
		THIP-LP	0.541
		MUSCIMOL-NP	0.863
		THIP-NP	0.879
	THIP-LP	SALINE-LP	*0.001
		MUSCIMOL-LP	0.541
		MUSCIMOL-NP	0.459
		THIP-NP	0.694
	MUSCIMOL-NP	SALINE-LP	*0.007
		MUSCIMOL-LP	0.863
		THIP-LP	0.459
		THIP-NP	0.766
	THIP-NP	SALINE-LP	*0.006
		MUSCIMOL-LP	0.879
		THIP-LP	0.694
		MUSCIMOL-NP	0.766

* *p* < 0.05.

### Experiment 2: GABA_A_R subunit gene expression in the SCN

**Figure 2.**
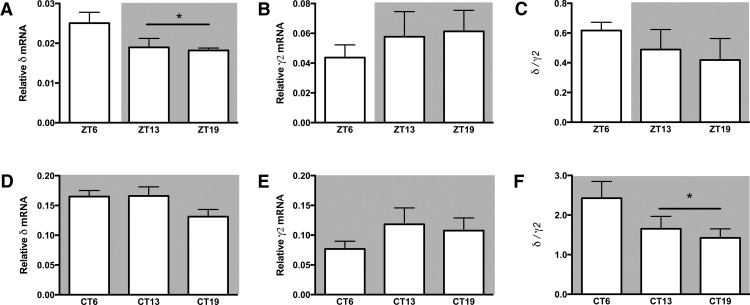
Rhythmic GABA_A_R mRNA expression in the SCN. Expression of the extrasynaptic GABA_A_δ receptor mRNA (***A***, ***D***) varied by circadian phase in a 14:10 LD cycle (***A***) with the highest level of expression during the day. Expression of the synaptic GABA_A_γ2 receptor RNA did not significantly vary across the day (***B***, ***E***). The ratio of extrasynaptic-to-synaptic receptor mRNA (δ:γ2) (***C***, ***F***) varied by circadian phase after 10 d in DD conditions (***F***), with the highest relative expression of GABA_A_δ occurring during the inactive phase (subjective day). *p ≤ 0.05. Statistics in Table 2.

When relative mRNA expression was analyzed by one-way ANOVA with time of day as the independent variable, variation in mRNA levels for both subunits did not reach statistical significance in either LD or DD (*p* > 0.05; Fig. 2; Table 2). However, when analyzed using an independent samples *t* test with circadian phase (light vs dark phase in LD; active vs inactive phase in DD) as the independent variable, differences in expression were apparent. The GABA_A_δ receptor subunit mRNA varied by circadian phase (i.e., ZT6 vs ZT13 and ZT19) in SCN dissections from hamsters housed under LD conditions (*t*_(15)_ = 2.498, *p* ≤ 0.05), with the highest expression during the light (inactive) phase (Fig. 2*A*). In contrast, the mRNA encoding the GABA_A_γ2 receptor subunit did not vary between the dark (active) and light (inactive) phases in LD (*t*_(15)_ = −0.979, *p* > 0.05; Fig. 2*B*). The ratio of the GABA_A_δ receptor subunit mRNA to the GABA_A_γ2 receptor subunit did not vary by circadian phase in LD (*t*_(15)_ = 1.181, *p* > 0.05; Fig. 2*C*). In hamsters housed in DD, the ratio of GABA_A_δ receptor subunit mRNA to GABA_A_γ2 receptor mRNA varied by circadian phase (i.e., CT6 vs CT13 and CT19) after 10 d in DD (*t*_(14)_ = 2. 317, *p* ≤ 0.05), with the highest ratio of GABA_A_δ-to-GABA_A_γ2 receptor subunit mRNA occurring during the inactive phase (Fig. 2*F*). There were no differences in GABA_A_δ receptor subunit mRNA or in GABA_A_γ2 receptor subunit mRNA in DD due to circadian phase (Fig. 2*D*,*E*).

**Table 2.** Analysis of GABA_A_ mRNA transcript expression

Condition	Type of test	Comparison	Gene	*F* statistic	*t* value	*p* value
LD	One-way ANOVA	Zeitgeber time	*δ*	(2,14) = 2.593		0.085
	One-way ANOVA	(ZT6 vs ZT13 vs ZT19)	*γ2*	(2,14) = 0.466		0.637
	One-way ANOVA		*δ:γ2*	(2,14) = 0.749		0.491
	Independent samples *t* test	Zeitgeber phase	*δ*		(15) = 2.498	*0.025
	Independent samples *t* test	(light vs dark)	*γ2*		(15) = −0.979	0.343
	Independent samples *t* test		*δ:γ2*		(15) = 1.181	0.256
DD	One-way ANOVA	Circadian time	*δ*	(2,13) = 2.598		0.112
	One-way ANOVA	(CT6 vs CT13 vs CT19)	*γ2*	(2,13) = 0.946		0.413
	One-way ANOVA		*δ:γ2*	(2,13) = 2.677		0.106
	Independent samples *t* test	Circadian phase	*δ*		(14) = 1.036	0.318
	Independent samples *t* test	(inactive vs active)	*γ2*		(14) = −1.734	0.191
	Independent samples *t* test		*δ:γ2*		(14) = 2.317	*0.036

* *p* < 0.05.

### Experiment 3: GABA_A_R subunit protein-IR in the SCN

**Figure 3.**
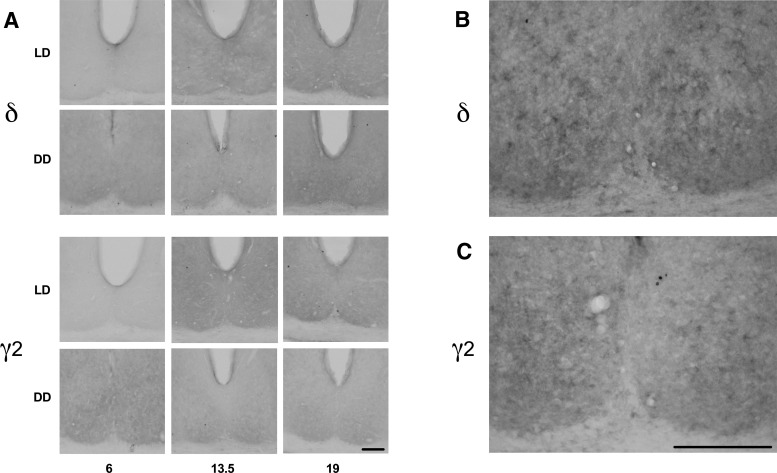
Photomicrographs of GABA_A_R subunit IR in the SCN. Representative photomicrographs of nickel-enhanced DAB immunohistochemical staining for extrasynaptic GABA_A_δ and synaptic GABA_A_γ2 proteins in the retinorecipient region (central posterior) of the SCN across time points and photic housing conditions (***A***). Representative 200× images of GABA_A_δ (***B***) and GABA_A_γ2 (***C***). Scale bars in ***A***, ***C*** = 150 μm.

Nickel-enhanced DAB immunohistochemistry revealed diffuse IR for both GABA_A_R subunit proteins throughout the SCN (Fig. 3). This diffuse staining pattern seen in the SCN has been previously reported for multiple GABA_A_R subunits in a variety of brain regions and neuronal cell types (Terai et al., 1998; Brunig et al., 2002; Crestani et al., 2002; Peng et al., 2004). As mentioned in Materials and Methods above, we measured and analyzed protein-IR in the whole SCN as well as in commonly used subdivisions of the SCN to allow the current results to be integrated with data from functional neuroanatomical subdivisions of the SCN that have been discussed previously (reviewed in Moore et al., 2002; Lee et al., 2003; Morin and Allen, 2006; Morin, 2007; Yan et al., 2007; Evans, 2016; Evans and Gorman, 2016; Albers et al., 2017).

**Figure 4.**
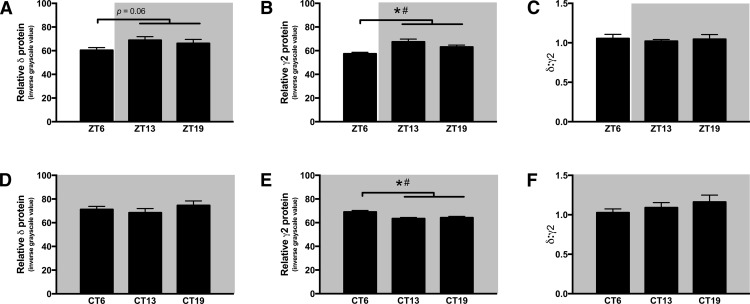
Rhythmic GABA_A_R subunit protein-IR in the SCN. Protein-IR of the synaptic GABA_A_γ2 receptor varied by circadian phase and CT in a 14:10 LD cycle (***B***), with the highest amount of protein-IR during the night (ZT13 and ZT19; active phase). However, after 10 d of free running in DD conditions, the rhythm of synaptic GABA_A_γ2 protein-IR was inverted with highest levels of protein-IR found during the subjective day (CT6, inactive phase; ***E***). The extrasynaptic GABA_A_δ receptor protein-IR did not vary by time of day or phase in either LD or DD conditions (***A***, ***D***). The ratio of δ-IR:γ2-IR did not significantly vary in the whole SCN in either LD or DD (***C***, ***F***). *p ≤ 0.05 active versus inactive phase; #*p* ≤ 0.05 ANOVA. Statistics in Table 3.

First, to allow direct comparison with the analyses of mRNA expression data in experiment 2, we performed a quantitative analysis of protein-IR of the whole SCN by one-way ANOVA with protein-IR as the dependent variable and zeitgeber time (LD) or circadian time (DD) as independent variables. We also analyzed whole SCN protein-IR using an independent samples *t* test with circadian phase (light vs dark phase in LD; active vs inactive phase in DD) as the independent variable as in experiment 2 above. Combining the two night time measurements and directly comparing them to the day time represents a functional grouping based on the effects of GABA_A_-active drugs across the circadian cycle as described above (Smith et al., 1989; Huhman et al., 1995; Gillespie et al., 1996; Gillespie et al., 1997; Gillespie et al., 1999; Mintz et al., 2002; Novak and Albers, 2004; Ehlen et al., 2006; Biello, 2009). The results of both analyses are found in Table 3. The intensity of GABA_A_δ protein-IR did not vary across time points in hamsters housed in LD (i.e., ZT6 vs ZT13 vs ZT19; Fig. 4*A*) or in hamsters housed in DD (i.e., CT6 vs CT13 vs CT19; Fig. 4*D*), nor by phase in hamsters housed in DD (i.e., CT6 vs CT13 and CT19; Fig. 4*D*). There was, however, a trend for greater GABA_A_δ protein-IR in the dark (active) phase in hamsters housed in a LD cycle (*p* = 0.06; Fig. 4*A*). GABA_A_γ2 protein-IR varied by time point and by phase in hamsters housed in a LD cycle; protein-IR was at nadir during the day and peak levels occurred at night, with the highest levels in the early night (*p* ≤ 0.05; Fig. 4*B*). After free-running in DD for 10 d, a circadian rhythm in GABA_A_γ2 protein-IR in the SCN was observed, with significantly higher levels occurring during the subjective day (CT6) than during the subjective night (CT13 and CT19, *p* ≤ 0.05; Fig. 4*E*). Based on studies using genetic techniques in mice, GABA_A_ γ2 and δ appear to reciprocally regulate each other’s expression and insertion into the cell membrane, independent of receptor activity (Korpi et al., 2002; Wu et al., 2013). Although it was not possible to measure membrane bound subunits, we analyzed the relative ratio of GABA_A_δ:GABA_A_γ2 protein-IR as a measure of how the relative amounts of these two proteins vary in relation to each other across the day. The ratio of extrasynaptic:synaptic subunit protein-IR did not vary in the whole SCN in LD or DD (*p* > 0.05; Fig. 4*C*,*F*).

**Table 3.** Analysis of GABA_A_R protein-IR

Condition	Type of test	Comparison	Protein	*F* statistic	*t* value	*p* value
LD	One-way ANOVA	Zeitgeber time	*δ*	(2,9) = 2.271		0.159
	One-way ANOVA	(ZT6 vs ZT13 vs ZT19)	*γ2*	(2,8) = 8.318		*0.011
	One-way ANOVA		*δ:γ2*	(2,8) = 0.165		0.850
	Independent samples *t* test	Zeitgeber phase	*δ*		(10) = −2.081	**0.064**
	Independent samples *t* test	(light vs dark)	*γ2*		(9) = −3.444	*0.007
	Independent samples *t* test		*δ:γ2*		(9) = 0.449	0.664
DD	One-way ANOVA	Circadian time	*δ*	(2,12) = 0.849		0.452
	One-way ANOVA	(CT6 vs CT13 vs CT19)	*γ2*	(2,12) = 7.754		*0.011
	One-way ANOVA		*δ:γ2*	(2,9) = 0.976		0.413
	Independent samples *t* test	Circadian phase	*δ*		(10) = −0.085	0.933
	Independent samples *t* test	(inactive vs active)	*γ2*		(10) = 4.069	*0.002
	Independent samples *t* test		*δ:γ2*		(10) = −1.219	0.251

* *p* < 0.05.

**Figure 5.**
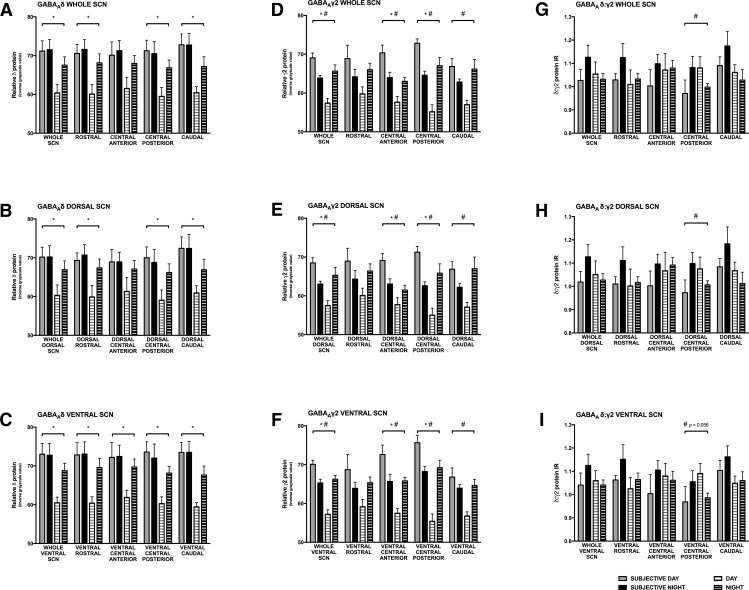
Interaction between lighting condition and circadian phase in GABA_A_R protein-IR in whole SCN and the dorsal and ventral subdivisions of the SCN. Lighting condition (LD vs DD) had a main effect on both extrasynaptic GABA_A_δ (***A–C***) and synaptic GABA_A_γ2 (***D–F***) protein-IR. There were no main effects of circadian phase (active phase vs inactive phase) on the protein-IR of either subunit. Lighting condition and circadian phase did not interact to affect protein-IR of extrasynaptic GABA_A_δ protein (***A–C***). There was an interaction of lighting condition and circadian phase in the protein-IR of synaptic GABA_A_γ2 receptors across the whole SCN and in all subdivisions, with the exception of the rostral SCN (***D–F***). Lighting condition and circadian phase interacted to affect the ratio of mean protein-IR of extrasynaptic GABA_A_δ to synaptic GABA_A_γ2 in the retinorecipient (central posterior) region of the SCN (***G***), where extrasynaptic receptor protein-IR was relatively higher during the subjective night and synaptic receptor protein-IR was relatively higher during the subjective day. This effect was significant in the dorsal central posterior SCN (***H***) and almost reached significance in the ventral central posterior SCN (***I***). *p ≤ 0.05 LD versus DD, #*p* ≤ 0.05 for interaction between lighting regimen and circadian phase. Statistics in Table 4.

Next, to determine whether there were phase-specific effects of lighting condition on protein-IR, we analyzed the SCN for both proteins of interest using a 2 × 2 MANOVA with grayscale value as the dependent variable and circadian phase (active vs inactive phase; i.e., ZT13, ZT19, CT13, CT19 vs ZT6, CT6) and lighting condition (LD vs DD) as independent variables. Grayscale values from the animal’s active phase represented the average of IR intensities across active time points, e.g., ZT13, ZT19, CT13, CT19, and inactive phase values were averages of IR intensities from ZT6 and CT6. We further analyzed the relationships between GABA_A_δ and GABA_A_γ2 subunit protein-IR by dividing the SCN into four regions along the rostral-caudal axis (LeSauter et al., 2002; Hamada et al., 2004), and into dorsal and ventral regions (Moore et al., 2002; Yan et al., 2007) as described in Materials and Methods. Statistics for this analysis are found in Table 4. There was a main effect for lighting condition; differences in both GABA_A_δ and GABA_A_γ2 protein-IR were observed between groups housed in LD versus DD in most subregions of the SCN with higher protein-IR in hamsters housed in DD (Fig. 5*A–F*). In contrast to the effects of lighting condition, there was no main effect for circadian phase; no differences in GABA_A_δ and GABA_A_γ2 protein-IR were observed in any of the subregions between the light and dark phase in hamsters housed in LD cycles or between the subjective day and night in hamsters housed in DD (Fig. 5*A–F*). No interactions were observed between lighting condition and circadian phase in the extrasynaptic GABA_A_δ protein-IR in any SCN subregion (*p* > 0.05 for all regions; Fig. 5*A–C*). However, there was an interaction between lighting condition and phase in GABA_A_γ2 protein-IR across the whole SCN and in all subregions, with the exception of the rostral SCN (*p* ≤ 0.05; Fig. 5*D–F*). Interestingly, an interaction in the ratio of GABA_A_δ:GABA_A_γ2 protein-IR between lighting condition and circadian phase was observed only in the central posterior SCN subregion (*F*_(1,1)_ = 4.72, *p* ≤ 0.05; Fig. 5*G*), which is the retinorecipient region in Syrian hamsters (LeSauter et al., 2002; Fig. 3*A*). Further analysis revealed that this interaction in the ratio of GABA_A_δ:GABA_A_γ2 protein-IR between lighting condition and circadian phase was significant in the dorsal central posterior subregion (*F*_(1,1)_ = 4.81, *p* ≤ 0.05; Fig. 5*H*), and nearly reached significance in the ventral central posterior subregion (*F*_(1,1)_ = 4.13, *p* = 0.056; Fig. 5*I*).

**Table 4.** MANOVA of GABA_A_R protein-IR in the SCN by region

SCN region		GABA_A_δ			GABAAγ2		GABA_A_δ:GABAAγ2		
	MS	*F*	*p*	MS	*F*	*p*	MS	*F*	*p*
Whole									
Phase	83.68	1.78	0.196	11.78	1.28	0.271	0.01	0.62	0.442
Light cycle	323.91	6.88	*0.015	126.77	13.81	*0.001	0.01	0.47	0.501
Phase × light cycle	68.48	1.45	0.240	235.19	25.62	*0.000	0.02	1.56	0.226
Dorsal									
Phase	73.46	1.51	0.231	7.20	0.56	0.465	0.01	0.68	0.421
Light cycle	272.70	5.61	*0.027	99.44	7.67	*0.012	0.01	0.43	0.518
Phase × light cycle	55.82	1.15	0.295	228.06	17.59	*0.000	0.02	1.67	0.212
Ventral									
Phase	106.46	2.30	0.143	23.07	3.51	0.077	0.01	0.52	0.479
Light cycle	426.95	9.23	*0.006	185.34	28.19	*0.000	0.01	0.51	0.483
Phase × light cycle	95.68	2.07	0.164	248.53	37.80	*0.000	0.02	1.56	0.226
Rostral									
Phase	122.54	2.68	0.115	5.76	0.16	0.690	0.02	1.32	0.265
Light cycle	287.92	6.30	*0.020	55.32	1.58	0.224	0.02	1.31	0.267
Phase × light cycle	72.96	1.60	0.219	116.86	3.33	0.084	0.01	0.44	0.516
Central anterior									
Phase	86.75	1.68	0.208	0.00	0.00	0.997	0.01	0.61	0.446
Light cycle	209.11	4.05	**0.056**	235.51	15.56	*0.001	0.00	0.03	0.855
Phase × light cycle	41.01	0.79	0.382	160.48	10.60	*0.004	0.01	0.46	0.505
Central posterior									
Phase	65.06	1.16	0.292	17.33	0.98	0.335	0.00	0.14	0.710
Light cycle	351.60	6.28	*0.020	260.17	14.67	*0.001	0.00	0.01	0.930
Phase × light cycle	97.42	1.74	0.200	581.64	32.80	*0.000	0.06	4.72	*0.043
Caudal									
Phase	66.28	1.13	0.298	51.62	2.71	0.116	0.00	0.13	0.718
Light cycle	475.70	8.12	*0.009	37.50	1.97	0.177	0.04	2.40	0.138
Phase × light cycle	68.66	1.17	0.290	189.02	9.92	*0.005	0.02	0.86	0.366
Dorsal rostral									
Phase	112.46	2.64	0.118	7.90	0.20	0.664	0.02	1.13	0.302
Light cycle	231.78	5.45	*0.029	45.00	1.11	0.305	0.02	1.13	0.300
Phase × light cycle	58.72	1.38	*0.252	120.19	2.97	0.101	0.01	0.61	0.444
Dorsal central anterior									
Phase	67.67	1.33	0.261	3.17	0.20	0.661	0.02	0.85	0.368
Light cycle	161.21	3.16	0.089	233.85	14.66	*0.001	0.00	0.14	0.710
Phase × light cycle	33.37	0.65	0.427	111.93	7.02	*0.016	0.01	0.29	0.595
Dorsal central posterior									
Phase	67.79	1.14	0.297	6.02	0.28	0.605	0.01	0.45	0.512
Light cycle	306.71	5.15	*0.033	181.46	8.36	*0.009	0.00	0.01	0.930
Phase × light cycle	82.33	1.38	0.252	554.05	25.52	*0.000	0.06	4.81	*0.041
Dorsal caudal									
Phase	52.12	0.76	0.392	52.56	2.02	0.171	0.00	0.09	0.772
Light cycle	425.19	6.21	*0.020	19.51	0.75	0.397	0.05	2.05	0.169
Phase × light cycle	54.35	0.79	0.382	234.85	9.04	*0.007	0.03	1.28	0.273
Ventral rostral									
Phase	146.81	2.63	0.119	2.72	0.09	0.768	0.03	1.67	0.212
Light cycle	402.46	7.20	*0.013	76.96	2.53	0.128	0.03	1.45	0.243
Phase × light cycle	102.11	1.83	0.190	110.90	3.64	0.072	0.00	0.17	0.687
Ventral central anterior									
Phase	132.25	2.23	0.149	10.31	0.48	0.496	0.01	0.28	0.603
Light cycle	308.83	5.20	*0.032	238.55	11.16	*0.003	0.00	0.02	0.885
Phase × light cycle	56.96	0.96	0.338	271.83	12.71	*0.002	0.01	0.80	0.384
Ventral central posterior									
Phase	59.39	1.06	0.313	53.12	2.53	0.128	0.00	0.01	0.908
Light cycle	436.59	7.81	*0.010	440.73	21.00	*0.000	0.00	0.16	0.698
Phase × light cycle	130.97	2.34	0.140	633.84	30.20	*0.000	0.05	4.13	**0.056**
Ventral caudal									
Phase	98.98	2.22	0.150	49.92	3.90	0.063	0.00	0.34	0.569
Light cycle	581.86	13.07	*0.001	85.46	6.68	*0.018	0.04	3.19	0.090
Phase × light cycle	100.62	2.26	0.146	118.04	9.22	*0.007	0.00	0.12	0.736

**Figure 6.**
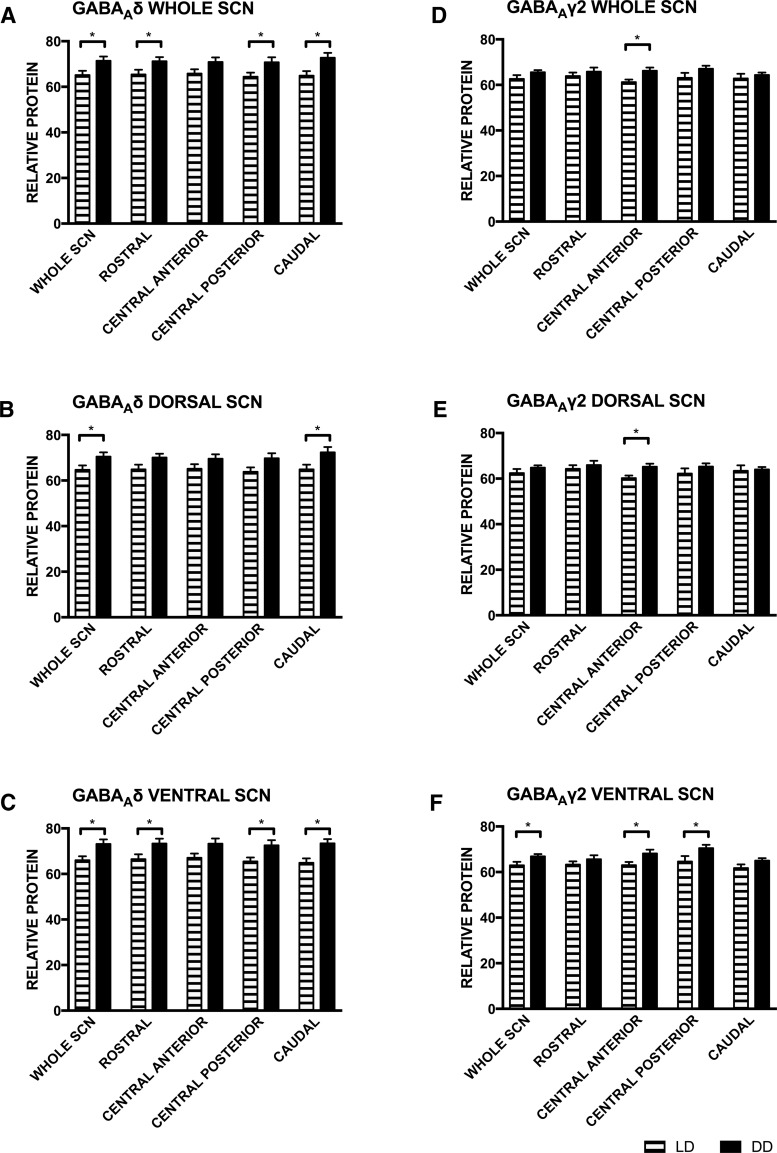
The SCN of hamsters housed in DD for 10 d had higher GABA_A_R subunit protein-IR than found in the SCN of those housed in LD (***A***, ***D***). The effects of housing in DD were more robust in the ventral SCN (***C***, ***F***) than the dorsal SCN (***B***, ***E***). Overall, protein-IR levels were calculated by averaging across the three sampling time points for each housing condition. *p ≤ 0.05 LD versus DD. Statistics in Table 5.

Given that we found a significant main effect of environmental lighting condition on protein-IR, we next analyzed our data to determine whether differences existed in protein-IR between LD and DD conditions. Protein-IR values, by SCN subdivision, were averaged across the day for animals in each lighting condition (i.e., LD: average of ZT6, ZT13, and ZT19; DD: average of CT6, 13, and 19), and then analyzed for effects of lighting condition (LD vs DD) by independent samples *t* test (Table 5). GABA_A_δ-IR was greater in DD than LD in many subregions of the SCN (Fig. 6), although the effects failed to reach statistical significance in several of the dorsal subregions and one of the ventral subregions (Fig. 6*B*,*C*). The effects of environmental light cycles were not as robust on GABA_A_γ2-IR, however, protein-IR levels were higher in DD in the central anterior region in the whole SCN and the dorsal SCN (Fig. 6*D*) as well as in the central anterior (Fig. 6*E*,*F*) and posterior ventral SCN (Fig. 6*F*).

**Table 5.** Independent samples *t* test comparing GABA_A_R protein-IR in regions of the SCN between LD and DD

Protein	SCN region	*t* value	*p* value
δ	Whole SCN	(25) = −2.314	*0.029
	Rostral	(25) = −2.137	*0.043
	Central anterior	(25) = −1.810	**0.082**
	Central posterior	(25) = −2.165	*0.040
	Caudal	(25) = −2.636	*0.014
γ2	Whole SCN	(21) = −1.544	0.142
	Rostral	(24) = −0.806	0.428
	Central anterior	(24) = −2.761	*0.011
	Central posterior	(23) = −1.582	0.127
	Caudal	(22) = −0.690	0.497
δ	Dorsal whole SCN	(25) = −2.117	*0.044
	Dorsal rostral	(25) = −1.996	**0.057**
	Dorsal central anterior	(25) = −1.606	0.121
	Dorsal central posterior	(25) = −1.981	**0.059**
	Dorsal caudal	(25) = −2.356	*0.027
γ2	Dorsal whole SCN	(21) = −1.203	0.242
	Dorsal rostral	(24) = −0.672	0.508
	Dorsal central anterior	(24) = −2.799	*0.010
	Dorsal central posterior	(23) = −1.160	0.258
	Dorsal caudal	(22) = −0.239	0.813
δ	Ventral whole SCN	(25) = −2.615	*0.015
	Ventral rostral	(25) = −2.277	*0.032
	Ventral central anterior	(25) = −2.036	**0.052**
	Ventral central posterior	(25) = −2.371	*0.026
	Ventral caudal	(25) = −3.176	*0.004
γ2	Ventral whole SCN	(21) = −2.178	*0.041
	Ventral rostral	(24) = −1.022	0.317
	Ventral central anterior	(24) = −2.283	*0.032
	Ventral central posterior	(23) = −2.146	*0.043
	Ventral caudal	(22) = −1.745	0.095

>* *p* < 0.05.

**Figure 7.**
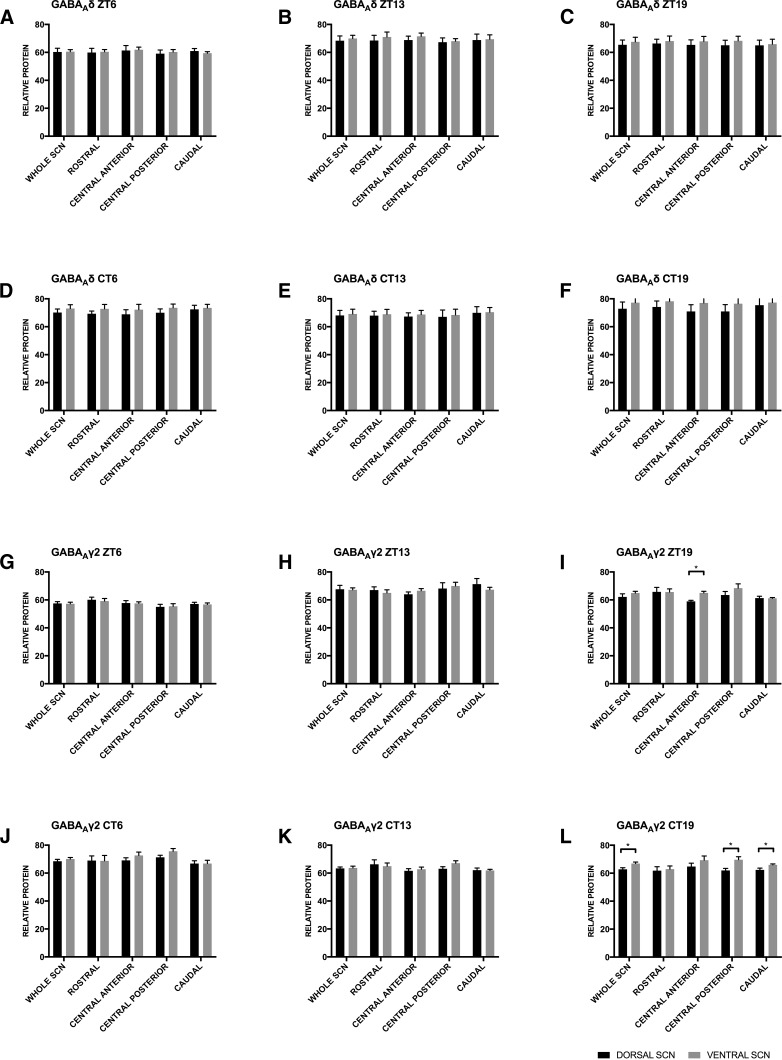
GABA_A_-TONIC receptor protein-IR did not vary between the dorsal and ventral SCN at any time point in either LD or DD (***A–F***). In the central anterior region, GABA_A_-PHASIC receptor protein-IR (***G–L***) was higher in the ventral SCN compared with the dorsal SCN at ZT19 (***I***). At CT19 GABA_A_-PHASIC receptor protein-IR was higher in the ventral SCN compared with the dorsal SCN across the whole SCN (***L***). This effect was strongest in the central posterior and caudal SCN. *p ≤ 0.05. Statistics in Table 6.

As discussed above the dorsal and ventral SCN have been shown to have different roles in entrainment (reviewed in Moore et al., 2002; Lee et al., 2003; Yan et al., 2007; Albers et al., 2017), thus we then analyzed our data to identify differences in GABA_A_R-IR between the dorsal and ventral SCN using an independent samples *t* test. The results of this analysis are found in Figure 7 and Table 6. We found no differences in GABA_A_δ protein-IR levels between the dorsal and ventral SCN at any time point in LD or DD (Fig. 7*A–F*). Compared with the dorsal region, the ventral SCN had higher levels of GABA_A_γ2 protein-IR late in the active phase (Fig. 7*I*,*L*). This effect was driven by higher protein-IR in the ventral central anterior region during the night in LD (ZT19; Fig. 7*I*) and by higher protein-IR in the ventral central posterior region during the subjective night in DD (CT19; Fig. 7*L*).

**Table 6.** Comparison of GABA_A_R protein-IR between dorsal and ventral SCN by time point

Protein	Time point	SCN region	*t* value	*p* value
δ	ZT6	Whole SCN	(6) = −0.066	0.095
		Rostral	(6) = −0.152	0.884
		Central anterior	(6) = −0.139	0.896
		Central posterior	(6) = −0.381	0.716
		Caudal	(6) = 0.692	0.515
δ	ZT13	Whole SCN	(6) = −0.398	0.704
		Rostral	(6) = −0.489	0.642
		Central anterior	(6) = −0.743	0.486
		Central posterior	(6) = −0.197	0.850
		Caudal	(6) = −0.111	0.915
δ	ZT19	Whole SCN	(6) = −0.443	0.673
		Rostral	(6) = −0.370	0.724
		Central anterior	(6) = −0.506	0.631
		Central posterior	(6) = −0.656	0.536
		Caudal	(6) = −0.172	0.869
δ	CT6	Whole SCN	(8) = −0.758	0.470
		Rostral	(8) = −0.948	0.371
		Central anterior	(8) = −0.660	0.528
		Central posterior	(8) = −0.930	0.380
		Caudal	(8) = −0.270	0.794
δ	CT13	Whole SCN	(8) = −0.210	0.839
		Rostral	(8) = −0.205	0.843
		Central anterior	(8) = −0.363	0.726
		Central posterior	(8) = −0.206	0.842
		Caudal	(8) = −0.082	0.937
δ	CT19	Whole SCN	(6) = −0.642	0.545
		Rostral	(6) = −0.642	0.544
		Central anterior	(6) = −0.875	0.415
		Central posterior	(6) = −0.712	0.503
		Caudal	(6) = −0.236	0.821
γ2	ZT6	Whole SCN	(6) = 0.162	0.877
		Rostral	(6) = 0.347	0.741
		Central anterior	(6) = 0.125	0.905
		Central posterior	(6) = −0.145	0.889
		Caudal	(6) = 0.197	0.850
γ2	ZT13	Whole SCN	(6) = 0.127	0.903
		Rostral	(6) = 0.623	0.556
		Central anterior	(6) = −1.115	0.308
		Central posterior	(6) = −0.368	0.726
		Caudal	(6) = 0.910	0.413
γ2	ZT19	Whole SCN	(4) = −1.076	0.343
		Rostral	(6) = 0.034	0.974
		Central anterior	(6) = −4.497	^*^0.004
		Central posterior	(6) = −1.240	0.261
		Caudal	(4) = 0.106	0.156
γ2	CT6	Whole SCN	(6) = −0.931	0.388
		Rostral	(8) = 0.041	0.969
		Central anterior	(8) = −1.151	0.283
		Central posterior	(6) = −1.796	0.123
		Caudal	(8) = 0.018	0.986
γ2	CT13	Whole SCN	(6) = −0.217	0.836
		Rostral	(8) = 0.347	0.738
		Central anterior	(8) = −0.519	0.618
		Central posterior	(8) = −1.708	0.127
		Caudal	(6) = 0.085	0.935
γ2	CT19	Whole SCN	(6) = −2.620	^*^0.040
		Rostral	(6) = −0.207	0.843
		Central anterior	(6) = −1.170	0.286
		Central posterior	(6) = −3.037	^*^0.023
		Caudal	(6) = −2.514	^*^0.046

* *p* < 0.05.

**Table 7.** Sample sizes

Experiment	Group	*N*
1	CT6 SALINE-NP	7
	CT6 THIP-NP	8
	CT6 MUSCIMOL-NP	9
	CT13.5 SALINE-LP	3
	CT13.5 THIP-LP	6
	CT13.5 MUSCIMOL-LP	5
	CT13.5 THIP-NP	3
	CT13.5 MUSCIMOL-NP	4
	CT19 SALINE-LP	6
	CT19 THIP-LP	4
	CT19 MUSCIMOL-LP	5
	CT19 THIP-NP	3
	CT19 MUSCIMOL-NP	4
2	CT6	5
	CT13.5	5
	CT19	6
	ZT6	6
	ZT13.5	6
	ZT19	5
3	CT6	5
	CT13.5	5
	CT19	5
	ZT6	4
	ZT13.5	4
	ZT19	4

## Discussion

The different temporal patterns in the expression of δ and γ2 subunit mRNA and protein-IR observed across all subregions of the SCN suggests that GABA_A_-TONIC extrasynaptic receptors and GABA_A_-PHASIC synaptic receptors are differentially regulated within the SCN. Interestingly, while δ protein-IR levels did not significantly change across the circadian cycle, γ2 protein-IR displayed significant rhythmicity in the SCN of hamsters housed in LD and DD. Comparison of the relative changes in γ2 protein-IR in hamsters housed in LD and DD suggests that this protein may be regulated by the circadian pacemaker as well as by environmental light. In hamsters housed in DD, the relative amounts of γ2 protein-IR varied significantly over the circadian cycle with peak levels occurring during the subjective day (Fig. 4*E*). In hamsters housed in LD, the amounts of γ2 protein-IR also varied significantly, however, the lowest levels of γ2 protein-IR were observed during light phase (Fig. 4*B*) suggesting that environmental light inhibits γ2 protein levels. The possibility that δ protein levels are also inhibited by light cannot be excluded because the lower levels of this protein-IR seen during the light phase in LD approached but did not reach statistical significance (Fig. 4*A*). Additionally, hamsters housed in LD, compared with those housed in DD, had reduced protein-IR for both subunits, and this effect was strongest in the ventral SCN (Fig. 6). Taken together, these data suggest that when analyzed across the entire SCN GABA_A_Rs containing the δ subunit (i.e., extrasynaptic GABA_A_-TONIC receptors) remain relatively constant across time whereas GABA_A_Rs containing the γ2 subunit (i.e., synaptic GABA_A_-PHASIC receptors) are regulated by the circadian pacemaker, and both receptor subtypes may be influenced by environmental lighting conditions. Of course, the presence of GABA_A_R subunits alone does not indicate the presence of functional receptors (Olsen and Sieghart, 2008), so direct measures of tonic and phasic currents within neurons of the SCN across the circadian cycle will be necessary to further support this possibility.

Despite the significant temporal variations in γ2 protein-IR in the SCN of hamsters housed in DD and LD the ratio of the mean protein-IR levels of δ-to-γ2 did not change significantly when analyzed across all subdivisions of the nucleus. In contrast, however, the ratio of δ-to-γ2 protein-IR was found to change significantly across the circadian cycle in a region-specific manner. Specifically, the ratio of δ-to-γ2 protein-IR was significantly greater during the night than during the day but only in the subregion of the SCN that corresponds to the retinorecipient area of the nucleus (Fig. 5). These data suggest that within the discrete region of the SCN that is innervated by direct projections from the retina, the δ subunit containing extrasynaptic GABA_A_-TONIC receptors may play a larger role in mediating the response to GABA than the γ2 containing synaptic GABA_A_-PHASIC receptors during the night, while the opposite is true during the day. If GABA_A_Rs in the retinorecipient region of the SCN mediate the ability of GABA to alter circadian phase, then GABA agonists that act selectively on extrasynaptic GABA_A_-TONIC receptors would be predicted to be more efficacious in modulating phase shifts during the night while agonists that act selectively on synaptic GABA_A_-PHASIC receptors would be predicted to be more effective during the day. The data from experiment 1, along with previous work (Ehlen and Paul, 2009; McElroy et al., 2009), support this hypothesis. Injection of THIP, an extrasynaptic δ superagonist, inhibits the phase shifting effects of light at night but has no effect on circadian phase during the day, and diazepam, a benzodiazepine that acts on γ2 subunit containing GABA_A_Rs, phase shifts circadian rhythms during the day but does not influence circadian phase at night. Further, muscimol, which activates both extrasynaptic and synaptic GABA_A_Rs, influences circadian phase during both the day and night. These studies, however, should be interpreted with caution because the pharmacological actions of these drugs can be complicated (for a review, see Albers, et al., 2017), and there may be differences in processes downstream from GABA_A_ signaling in the SCN which also influence the behavioral responses to GABA_A_R activation across the circadian cycle. Nevertheless, the significant increase in the ratio of δ-to-γ2 receptor protein-IR within the retinorecipient region of the SCN during the subjective night could indicate a shift in the balance of GABA’s effects from synaptic phasic modulation during the subjective day to extrasynaptic tonic modulation during the subjective night.

Other recent data also suggest that rhythms in the balance of tonic versus phasic GABA_A_-induced conductance may be important in determining the phase of the circadian pacemaker. It has recently been demonstrated that the *sustained* activation of GABA_A_Rs in the SCN (>4 h) is both necessary and sufficient for the induction of phase delays by light (Hummer et al., 2015). Interestingly, recent SCN modeling studies predict that sustained tonic GABA signaling, but not a sustained phasic GABA signaling, can phase shift the molecular pacemaker (DeWoskin et al., 2015). These data combined with the present findings that the ratio of tonic:phasic GABA_A_Rs may be highest during the subjective night within the retinorecipient subregion of the nucleus suggest the hypothesis that the sustained effects of GABA on phase resetting at night may be mediated by extrasynaptic GABA_A_-TONIC receptors. Thus, a sustained tonic GABA signal may necessarily need to be transduced through a nondesensitizing receptor, such as the extrasynaptic GABA_A_-TONIC receptor. Additional experiments will be necessary to determine which GABA_A_R subtype mediates the sustained effects of GABA on photic phase shifts, or whether both tonic and phasic receptors play a role in this intriguing process.

Data on GABA_A_R mRNA expression in the SCN are sparse in the literature. Using Northern blottings in extracts of the SCN from mice, transcripts were found for α_1,2,3,4,5_, β_1,2,3_, and γ_1,2_ subunits, however, transcripts for the δ and ρ subunits were not detected (O'Hara et al., 1995). Using microarray technology, transcripts for all 19 currently identified GABA_A_R subunits were found in the SCN of mice (Mouse 1.OST SCN 2014; Pizarro et al., 2013). It is interesting to note that within this same database in another dataset (mouse wild-type SCN, GNF Microarray), there was a diurnal rhythm in γ2 mRNA expression in the SCN of wild-type mice, with peak expression at night and nadir during the day. Interestingly, this expression pattern was antiphase in *clock* mutants with γ2 mRNA peak expression occurring during the day (Pizarro et al., 2013), suggesting that transcription of γ2 may be under control of one of the genes comprising the molecular circadian pacemaker (i.e., *clock*).

Studies on GABA_A_ subunit protein expression in the SCN are also quite limited. Gao and colleagues investigated the protein expression of six different GABA_A_R subunits in the SCN of rats and found that IR was robust for α_2_, α_3_, α_5_, and γ_2_, but no staining was detected for α_1_ and β_2/3_ (Gao et al., 1995). However, this neuroanatomical study did not indicate the time of day the tissues were collected. Given that GABA_A_R subunit protein can vary considerably across the circadian cycle (Fig. 3; Naum et al., 2001), it is possible that the lack of IR reported for α_1_ and β_2/3_ was an artifact of time of day the tissues were collected. Indeed, both α_1_ and β_2/3_ mRNA expression has been reported in the SCN (O'Hara et al., 1995; Pizarro et al., 2013), as well as β_3_ protein (Naum et al., 2001; Belenky et al., 2003). To our knowledge, only one previous study has directly investigated temporal patterns of GABA_A_R protein expression in the SCN. Of the four subunits examined (α_2_, α_5_, β_1_, β_3_), only β_1_ was found to vary across the circadian cycle, with more protein at night (ZT16 and CT16) than during the day (ZT4 and CT4; Naum et al., 2001). Given that tissues were collected after only 2 d in DD, it is not clear whether this is a true circadian rhythm or a damped rhythm following exposure to the 14:10 LD cycle. As noted earlier, the presence of GABA_A_ subunits does not necessarily demonstrate the existence of functional GABA_A_Rs containing those subunits (reviewed in Olsen and Sieghart, 2008). A pharmacological study of Zn^2+^-mediated GABA_A_R inhibition found greater inhibition of GABA-induced current during the day than at night in the SCN of rats housed in standard LD conditions (Kretschmannova et al., 2003). Given that GABA_A_Rs with a γ subunit are insensitive to Zn^2+^ inhibition, the authors concluded that the proportion of γ subunit containing receptors in the SCN was higher at night than during the day, which is consistent with our current findings in the SCN of hamsters housed in LD (Figs. 4, 5).

Our current findings that protein-IR patterns for GABA_A_Rs in the SCN are different from the expression patterns of their genes (Figs. 2, 4) is a phenomenon that has also been reported in other studies (described below) on transcript-protein expression relationships in the SCN. Peroxisome proliferator-activated receptor β/δ mRNA and protein display rhythmicity in the SCN of animals housed in LD cycles, but in DD, mRNA expression remains rhythmic whereas protein expression does not (Challet et al., 2013). Further evidence that transcript and protein rhythms can be uncoupled comes from a recent SCN proteome study that analyzed 2112 proteins. This study concluded that “transcript levels are a poor predictor of protein abundance” based on the finding that among 421 transcripts which were expressed in a 24 h pattern, only nine of the proteins corresponding to those transcripts were rhythmically expressed (Chiang et al., 2014). Taken together, these findings suggest that the circadian protein rhythms of GABA_A_Rs subunits and their ratios in the SCN are more likely to be regulated by posttranscriptional factors than by transcriptional rhythms.

How might rhythms in protein expression and relative ratios of proteins develop independent of rhythms (or lack thereof) in transcripts? One possibility is that homeostatic reciprocal regulation between GABA_A_δ and GABA_A_γ2 proteins may affect their expression in a seesaw manner (Korpi et al., 2002; Wu et al., 2013), resulting in the different effects of tonic and phasic GABA_A_ agonists across the circadian cycle in the SCN. This mechanistically simple hypothesis does not appear to be supported by our data across the whole SCN, as changes in GABA_A_γ2 protein-IR are not accompanied by significant and reciprocal changes in GABA_A_δ protein-IR (Fig. 4). Indeed, lighting conditions (LD vs DD) appear to have a greater influence on the expression of GABA_A_Rs than homeostatic competition driven by their relative abundance (Figs. 4–6). The interaction of light and circadian phase on GABA_A_R ratio in the retinorecipient SCN (Fig. 5*G–I*) does suggest that protein expression in this area may be differentially regulated than in other SCN regions. Thus, it may be possible that homeostatic reciprocal regulation between GABA_A_δ and GABA_A_γ2 protein may indeed occur in the retinorecipient SCN.

In conclusion, circadian rhythms in the ratio of δ-to-γ2 GABA_A_R-IR in the retinorecipient SCN may mediate the phase-dependent effects of GABA on the circadian pacemaker. Within the circadian pacemaker, patterns of GABA_A_R transcript expression do not predict patterns of protein expression, and light appears to have a greater influence on GABA_A_R protein expression than does circadian transcriptional regulation. Although the effects of environmental light on GABA_A_R protein-IR are apparent across the entire SCN, the retinorecipient area is differentially affected. These findings provide insight into the complex effects of GABA in the SCN across the circadian cycle and highlight the need for future studies to identify the exact subunit composition, anatomic distribution, temporal patterns of expression, and regulatory factors influencing the expression and function of GABA_A_Rs in the circadian pacemaker.

## Synthesis

Reviewing Editor: Rae Silver, Columbia University Decisions are customarily a result of the Reviewing Editor and the peer reviewers coming together and discussing their recommendations until a consensus is reached. When revisions are invited, a fact-based synthesis statement explaining their decision and outlining what is needed to prepare a revision will be listed below. The following reviewer(s) agreed to reveal their identity: Charles Allen, Rebecca Prosser

As previously noted, the research indicates that two GABA(A) receptor subunits are expressed throughout the suprachiasmatic nucleus. The data also suggest that there is a small rhythm in the expression of the subunits that might contribute to different roles for synaptic and extrasynaptic GABA(A) receptors in the SCN. The authors have been responsive to prior reviews, and at this point some small revisions are suggested. These will be reviewed by the editor (only).

The manuscript tends to overstate the significance of the data, given that the rhythms shown, while statistically significant, are small relative to the total protein/mRNA levels. This can be clarified by changing the visual abstract to reflect a less dramatic day/night difference in the abundance of the two subunits. The significance statement and abstract discuss these differences as a change in balance, which is well put and more accurate. Also, the discussion might state directly that combining the two nighttime data sets and comparing them to the daytime reflects a functional grouping and is reasonable, and that it was necessary because of the subtle rhythms being measured. That said, given that the effects are subtle, this may suggest that the role of the two populations of receptors in the behavioral responses is more related to their downstream signaling (among other possible mechanisms) than to changes in their expression pattern?

Please address these comments; the changes will be reviewed by the editor only.
